# LEADER 7: cardiovascular risk profiles of US and European participants in the LEADER diabetes trial differ

**DOI:** 10.1186/s13098-016-0153-5

**Published:** 2016-06-02

**Authors:** Guy E. H. M. Rutten, Cees J. Tack, Thomas R. Pieber, Abdurrahman Comlekci, David Dynnes Ørsted, Florian M. M. Baeres, Steven P. Marso, John B. Buse, on behalf of the LEADER Investigators

**Affiliations:** Julius Center for Health Sciences and Primary Care, University Medical Center Utrecht, STR 6.131, P.O. Box 85500, 3508 GA Utrecht, The Netherlands; Department of Internal Medicine, Radboud University Medical Center, Nijmegen, The Netherlands; Division of Endocrinology and Metabolism, Medical University of Graz, Graz, Austria; Division of Endocrinology, Dokuz Eylul University Medical School, Inciralti, Izmir, Turkey; Novo Nordisk, Søborg, Denmark; Department of Internal Medicine, UT Southwestern, Dallas, TX USA; Department of Medicine, The University of North Carolina School of Medicine, Chapel Hill, NC USA

**Keywords:** Type 2 diabetes, External validity, Generalizability, Cardiovascular outcome trial, Heterogeneity

## Abstract

**Aims:**

To determine whether US and European participants in the Liraglutide Effect and Action in Diabetes: Evaluation of cardiovascular outcome Results (LEADER) trial differ regarding risk factors for cardiovascular mortality and morbidity.

**Methods:**

Baseline data, stratified for prior cardiovascular disease (CVD), were compared using multivariable logistic regression analysis to establish whether region is an independent determinant of achieved targets for glycated hemoglobin (HbA1c), blood pressure (BP), and low-density lipoprotein (LDL)-cholesterol.

**Results:**

Independent of CVD history, US participants were more often of non-White origin and had a longer history of type 2 diabetes, higher body weight, and higher baseline HbA1c. They had substantially lower systolic and diastolic BP, and a marginally lower LDL-cholesterol level. Fewer US participants were diagnosed with left ventricular dysfunction. In the largest group of patients, those with prior CVD and the highest cardiovascular risk, US participants were more often female, had a higher waist circumference, and had a decreased estimated glomerular filtration rate, but less frequently prior myocardial infarction or angina pectoris.

**Conclusions:**

There were baseline differences between US and European participants. These differences may result from variation in regional targets for cardiovascular risk factor management, and should be considered in the analysis and reporting of the trial results.

*Clinical trial identifier: ClinicalTrials.gov, NCT01179048*

## Background

The Liraglutide Effect and Action in Diabetes: Evaluation of cardiovascular outcome Results (LEADER) trial [1] is one of at least 16 cardiovascular outcome trials in type 2 diabetes (T2D) that will report before 2020 [2]. Driven by regulatory requirements to demonstrate cardiovascular safety, trial sample sizes are large. To recruit 10‒20,000 participants per trial, there are 17–27 countries per trial, resulting in a wider range of racial and ethnic participation, as well as greater variation in clinical practice patterns than in previous T2D studies [2]. The clinical usefulness of trial data obtained for a particular clinician or policy-maker depends on how well the trial population mirrors the patients in that clinician’s practice, or the population for whom the policy is developed. This external validity is determined by inclusion criteria, but also by patients’ enrolment, retention, and protocol adherence. The age distribution of trial participants in seven large diabetes trials often did not reflect that of a contemporary British T2D patient population [3]. Most ongoing diabetes cardiovascular outcome trials are being conducted in populations with high cardiovascular risk with a long history of T2D [2]. Women and ethnic minorities are under-represented in US-based clinical trials, although these groups have disproportionally higher rates of chronic diseases [4]. The generalizability of results even from well-performed trials may be jeopardized when participants differ from the clinical population with respect to attributes that modify treatment [5]. In a multivariate model, female sex, renal insufficiency, and heart failure were significant predictors of not participating in a trial [6]. Such clinical factors are important because they may modify treatment effects. The table of baseline characteristics in trial reports should focus on baseline factors associated with primary outcomes [7].

Patient enrolment is likely to be dependent on the specific types of investigator practices (generalist, diabetologist, cardiologist, nephrologist, etc.) and on the healthcare systems they operate within. A UK study suggests that selection of study sites should aim to represent the general medical management of T2D patients [8]. It might be questioned whether such a strategy is economically and logistically possible in large global trials.

We seek to examine whether there are important baseline differences in cardiovascular risk markers among LEADER participants enrolled in the US and Europe. This study of these two populations was prompted by the substantial differences between the US and Europe, for example with respect to demographics; cultural norms regarding diet, activity, and other health behaviors; prevailing treatment guidelines for glycemia, blood pressure (BP), lipids, and cardiovascular management; and funding and nature of healthcare systems [9, 10]. We examined baseline demographic and clinical characteristics of LEADER participants in the US and Europe without a priori hypotheses. Such an exploratory investigation may generate hypotheses about possible heterogeneity [11], and be informative for potential post hoc analyses [11, 12].

## Methods

### Study design and participants

We used baseline data from LEADER (NCT01179048), a phase 3b, multicenter, international, randomized, double-blind, placebo-controlled clinical trial with long-term follow-up in 9340 patients with T2D. This trial will provide data on the cardiovascular safety of liraglutide compared to placebo. The trial design has been described in more detail previously [1]. Enrolment took place from September 2010 until April 2012. Patients with T2D who were either drug-naive or treated with oral blood glucose-lowering agents or selected insulin regimens (human neutral protamine Hagedorn, long-acting analog, or premixed) were eligible. LEADER enrolled two distinct populations of high-risk patients: (1) patients with prior cardiovascular disease (CVD), ≥50 years old (defined as having one or more of the following cardiovascular comorbidities: prior myocardial infarction, stoke, transient ischemic attack, arterial revascularization, chronic heart failure [New York Heart Association {NYHA} class II–III], or chronic renal failure); (2) patients without prior CVD, ≥60 years old with one or more cardiovascular risk factors: micro-albuminuria or proteinuria, hypertension and left ventricular hypertrophy by electrocardiogram (ECG) or imaging, or ankle-brachial index <0.9. Enrolment of approximately 400 patients with moderate (estimated glomerular filtration rate [eGFR] 30‒59 mL/min/1.73 m^2^) and 200 with severe (eGFR <30 mL/min/1.73 m^2^) renal impairment was also pre-specified [1].

The present analysis compares baseline data between US and European populations from LEADER, stratified by patients’ cardiovascular history. The European population includes all patients enrolled from Austria, Belgium, Czech Republic, Denmark, Finland, France, Germany, Greece, Ireland, Italy, the Netherlands, Norway, Poland, Romania, Serbia, Spain, Sweden, Turkey, and the UK. The trial was designed and conducted according to the declaration of Helsinki and approved by local ethical committees. All subjects signed informed consent before any study procedure was performed.

### Outcome measures

Baseline participant characteristics were recorded at screening visits (Table 1). Concomitant illness was any illness present at the start of the trial, including history of CVD (myocardial infarction, stroke, angina pectoris, arrhythmia, left ventricular dysfunction). Concomitant medication use was defined as use of any medication other than trial product at randomization. Data on blood glucose-lowering medication, the number and type of antihypertensive medication, statin use, other lipid-lowering medication, and aspirin were collected. Height, body weight, and waist circumference were measured without shoes and in light clothing. Systolic BP (SBP) and diastolic BP (DBP) were measured after at least 5 min rest in the sitting position, with the legs uncrossed and the back and arm supported. Blood samples were collected at randomization and assessed in a central laboratory for glycated hemoglobin (HbA1c), lipids, creatinine, and albumin:creatinine ratio (ACR).

**Table 1:** Baseline differences between Europe and US patients, stratified by history of prior CVD

	No prior CVD group (*n* = 1013)			Prior CVD group (*n* = 4996)		
	Europe (*n* = 599)	USA (*n* = 414)	*P* value	Europe (*n* = 2922)	USA (*n* = 2074)	*P* value
Gender, female (*n*, %)	223 (37.2)	177 (42.8)	0.077	857 (29.3)	710 (34.2)	0.001
Age, years (mean, SD) (*n*, %)	66.0 (5.0)	66.1 (5.6)	0.783	64.6 (7.5)	64.3 (7.7)	0.166
50–59	10 (1.7)	8 (1.9)		798 (27.3)	589 (28.4)	
60–69	454 (75.8)	310 (74.9)		1364 (46.7)	971 (46.8)	
70–79	127 (21.2)	84 (20.3)	0.355	694 (23.8)	451 (21.7)	0.240
80–89	8 (1.3)	12 (2.9)		65 (2.2)	62 (3.0)	
90–99	0 (0)	0 (0)		1 (0.0)	1 (0.0)	
Diabetes duration, years (mean, SD)	11.2 (6.8)	13.2 (7.9)	<*0.001*	11.7 (7.6)	13.5 (8.8)	*<0.001*
Race (*n*, %)						
Asian	3 (0.5)	9 (2.2)		25 (0.9)	37 (1.8)	
Black	2 (0.3)	101 (24.4)	<*0.001*	14 (0.5)	366 (17.6)	*<0.001*
White	592 (98.8)	295 (71.3)		2871 (98.3)	1622 (78.2)	
Other	2 (0.3)	9 (2.2)		12 (0.4)	49 (2.4)	
Body weight, kg (mean, SD)	94.0 (18.1)	98.9 (23.4)	<*0.001*	93.6 (18.6)	101.1 (21.8)	*<0.001*
BMI, kg/m^2^ (mean, SD) (*n*, %)	33.0 (5.6)	34.4 (6.9)	<*0.001*	32.7 (5.8)	34.6 (6.8)	*<0.001*
18–25	25 (4.2)	24 (5.8)		181 (6.2)	96 (4.6)	
25–30	176 (29.4)	91 (22.0)		843 (28.9)	438 (21.1)	
30–35	216 (36.1)	125 (30.2)	<*0.001*	1024 (35.0)	686 (33.1)	*<0.001*
35–40	113 (18.9)	91 (22.0)		554 (19.0)	478 (23.0)	
>40	69 (11.5)	83 (20.0)		316 (10.8)	375 (18.1)	
Waist circumference, cm (mean, SD)	112.2 (15.5)	114.1 (18.4)	0.072	111.2 (15.3)	114.5 (17.0)	*<0.001*
HbA1c, % (mean, SD)	8.4 (1.3)	8.9 (1.6)	<*0.001*	8.3 (1.3)	8.8 (1.5)	*<0.001*
HbA1c at target (*n*, %)						
≤7.5 %	186 (31.1)	68 (16.4)	<*0.001*	880 (30.1)	481 (23.2)	<*0.001*
≤7 %	49 (8.2)	18 (4.3)	0.016	264 (9.0)	157 (7.6)	0.066
SBP, mmHg (mean, SD)	144.1 (17.5)	135.5 (17.1)	<0.001	140.0 (18.4)	133.3 (18.5)	<*0.001*
DBP, mmHg (mean, SD)	81.0 (9.7)	76.0 (9.5)	<*0.001*	78.5 (10.2)	74.7 (10.5)	<*0.001*
BP at target (*n*, %) (Europe < 140/80 and USA 130/80 mmHg)	146 (24.4)	134 (32.4)	*0.005*	974 (33.3)	800 (38.6)	<*0.001*
LDL, mmol/L (mean, SD)	2.5 (0.9)	2.3 (0.9)	<*0.001*	2.3 (0.9)	2.2 (0.9)	0.113
At target (*n*, %)	319 (53.3)	273 (65.9)	<*0.001*	1934 (66.2)	757 (36.5)	<*0.001*
HDL, mmol/L (mean, SD)	1.3 (0.3)	1.2 (0.4)	0.104	1.2 (0.3)	1.2 (0.3)	*0.008*
Triglyceride, mmol/L (mean, SD)	2.0 (1.3)	2.0 (2.0)	0.749	2.1 (1.8)	2.0 (1.5)	0.038
Creatinine, μmol/L (mean, SD)	71.6 (14.6)	73.1 (15.6)	0.106	85.4 (31.2)	94.7 (38.2)	<*0.001*
ACR, mg/mmol (mean, SD)	10.6 (30.5)	14.8 (50.5)	0.135	16.4 (57.3)	26.5 (82.0)	<*0.001*
eGFR (mean, SD)	90.0 (24.0)	90.3 (20.5)	0.847	81.3 (26.8)	74.8 (27.1)	<*0.001*
Renal function, eGFR (mL/min/1.73 m^2)^ (*n*, %)						
Normal (≥ 90)	268 (44.7)	181 (43.7)		1079 (36.9)	591 (28.5)	
Mild decline (60–90)	331 (55.3)	233 (56.3)	0.748	1191 (40.8)	809 (39.0)	<*0.001*
Moderate decline (30–60)	0 (0.0)	0 (0.0)		605 (20.7)	625 (30.1)	
Severe decline (< 30)	0 (0.0)	0 (0.0)		47 (1.6)	49 (2.4)	
Micro-albuminuria (*n*, %)	246 (41.1)	233 (56.3)	<*0.001*	347 (11.9)	248 (12.0)	0.930
Macro-albuminuria (*n*, %)	31 (5.2)	65 (15.7)	<*0.001*	77 (2.6)	66 (3.2)	0.253
Myocardial infarction (*n*, %)	0 (0)	0 (0)		567 (19.4)	321 (15.5)	<*0.001*
Stroke (*n*, %)	0 (0)	0 (0)		434 (14.9)	289 (13.9)	0.363
Angina pectoris (*n*, %)	0 (0)	5 (0.8)	0.062	1114 (38.1)	617 (29.7)	<*0.001*
Arrhythmia (*n*, %)	57 (9.5)	45 (10.9)	0.482	479 (16.4)	394 (19.0)	0.017
Left ventricle dysfunction (*n*, %)	188 (31.4)	56 (13.5)	<*0.001*	702 (24.0)	379 (18.3)	<*0.001*
Smoking (*n*, %)						
Current	83 (13.9)	57 (13.8)		407 (13.9)	278 (13.4)	
Never	248 (41.4)	187 (45.2)	0.455	946 (32.4)	754 (36.4)	0.013
Previous	268 (44.7)	170 (41.1)		1569 (53.7)	1042 (50.2)	
Pre-trial treatment (*n*, %)						
None/diet	16 (2.7)	41 (9.9)		102 (3.5)	226 (10.9)	
Insulin only	19 (3.2)	22 (5.3)	<*0.001*	200 (6.8)	210 (10.1)	<*0.001*
OADs only	380 (63.4)	213 (51.4)		1624 (55.6)	973 (46.9)	
Insulin + OADs	184 (30.7)	138 (33.3)		996 (34.1)	665 (32.1)	
Number of oral blood glucose-lowering drugs (*n*, %)						
No OAD	219 (36.6)	201 (48.6)		1298 (44.4)	1101 (53.1)	
1 OAD	155 (25.9)	83 (20.0)	0.424	707 (24.2)	428 (20.6)	0.013
2 OADs	199 (33.2)	109 (26.3)		826 (28.3)	463 (22.3)	
>2 OADs	26 (4.3)	21 (5.1)		91 (3.1)	82 (4.0)	
BP-lowering medication (*n*, %)	517 (86.3)	360 (87.0)	0.767	2729 (93.4)	1930 (93.1)	0.639
Number of antihypertensive medications (*n*, %)						
0	82 (13.7)	54 (13.0)		193 (6.6)	144 (6.9)	
1	225 (37.6)	182 (44.0)	0.018	731 (25.0)	607 (29.3)	*0.001*
2	172 (28.7)	126 (30.4)		1315 (45.0)	920 (44.4)	
3	93 (15.5)	44 (10.6)		533 (18.2)	324 (15.6)	
4	27 (4.5)	8 (1.9)		150 (5.1)	79 (3.8)	
Statin (*n*, %)	362 (60.4)	298 (72.0)	<*0.001*	2369 (81.1)	1704 (82.2)	0.330
Other lipid-lowering drugs (*n*, %)	2 (0.3)	11 (2.7)	*0.001*	39 (1.3)	44 (2.1)	0.032
Aspirin (*n*, %)	192 (32.1)	198 (47.8)	<*0.001*	1934 (66.2)	1441 (69.5)	0.014

*ACR* albumin:creatinine ratio, *BMI* body mass index, *BP* blood pressure, *CVD* cardiovascular disease, *DBP* diastolic blood pressure, *eGFR* estimated glomerular filtration rate, *HDL* high-density lipoprotein, *LDL* low-density lipoprotein, *OAD* oral antidiabetic drug, *SBP* systolic blood pressure, *SD* standard deviation

*P* values are for difference between region (Europe vs. USA) on covariates (t test) and factors (Chi square)

###  Target levels

In 2010, ‘general’ HbA1c goals varied among European countries between <6.5 % and <7.0 % (<48 and <53 mmol/mol) [9]. The American Diabetes Association (ADA) recommended in 2010 that less stringent HbA1c goals than the general goal of 7.0 % (53 mmol/mol) may be appropriate for patients with advanced macrovascular complications [10]. An ADA/European Association for the Study of Diabetes position statement stated that HbA1c goals between 7.5 and 8.0 % (58 and 64 mmol/mol), or even slightly higher, are appropriate for patients with established vascular complications or extensive comorbid conditions [13]. Previously, SBP and DBP targets also differed among European countries, between <130 and 140 mmHg and <80 or <85 mmHg, respectively [9]. Until 2013, the ADA recommended a BP target of <130/80 mmHg; in 2014, US guidance on BP in diabetes was relaxed to <140/<80 mmHg [14]. Similarly, at the onset of the study in 2010, low-density lipoprotein (LDL)-cholesterol targets differed between European countries, ranging from <100 mg/dL (2.6 mmol/L) and 115 mg/dL (3 mmol/L) for primary prevention [9]. At that time, the LDL target for US participants was <100 mg/dL (<2.6 mmol/L) (no prior CVD) or <70 mg/dL (<1.8 mmol/L) (prior CVD) [10], with the latter goal termed ‘an option’ in the 2014 recommendations [14].

For the present study, we defined two different HbA1c targets: ≤7.0 % and ≤7.5 % (≤53 and ≤58 mmol/mol); for both CVD strata and both regions [15]. BP at target was defined as <140/<80 and <130/<80 mmHg for European and US participants, respectively. LDL-cholesterol at target was defined as <100 mg/dL (<2.6 mmol/L) for European participants and <100 mg/dL (<2.6 mmol/L) (no prior CVD group) or <70 mg/dL (<1.8 mmol/L) (prior CVD group) for US participants.

### Statistical analyses

All analyses were performed using SAS software (version 9.3). Continuous variables were reported as means with standard deviations, categorical variables as numbers and percentages. Because a patient’s history of CVD is likely to be related to the cardiovascular outcome of the LEADER trial, the analysis of differences between regions (US vs. Europe) was stratified by prior and no prior CVD. We used t-tests for continuous variables or Chi square test for class variables. Logistic regression analyses were then used to determine whether region (Europe/US) was an independent determinant of achieving target for HbA1c, BP, and LDL-cholesterol, respectively. These analyses were adjusted for several well-known possible confounding variables: age, sex, diabetes duration, race, pre-trial diabetes treatment, body mass index (BMI) category, and renal function (creatinine, ACR, and eGFR); these variables were included based on subject matter considerations and availability of data. See Tables 2, 3, 4 for details. *P* values ≤0.01 were considered statistically significant.

## Results

### Baseline differences between Europe and US patients: whole cohort

Irrespective of CVD history, US and European participants differed on several important CVD risk factors and other diabetes complications (Table 1). US participants were more often of non-White origin, had longer T2D duration, had higher body weight, and were more often in the highest BMI categories compared with European participants. Furthermore, US participants had a 0.5 % higher HbA1c and a different distribution of blood glucose-lowering treatments than European participants. Significantly fewer US participants were at HbA1c target ≤7.5 % (≤58 mmol/mol). However, US participants had substantially lower SBP and DBP, and marginally lower LDL-cholesterol level than European participants. Significantly more European than US participants were diagnosed with left ventricular dysfunction.

### Baseline differences between Europe and US patients: group without prior CVD

In the group without prior CVD, US participants were more frequently at BP target (with different target levels in both regions) and more frequently at LDL target (with comparable targets in both regions, but with significantly more US participants on lipid-modifying medications) than participants from Europe. In addition, more US than European participants without prior CVD had micro- or macro-albuminuria, but the percentages of these patients with decreased kidney function did not differ between regions (Table 1).

### Baseline differences between Europe and US patients: group with prior CVD

In the largest group of patients, those with prior CVD and the highest cardiovascular risk, US participants were more often female and had a higher waist circumference than participants from Europe. A higher percentage of US participants were at BP target and a lower percentage of US participants with prior CVD attained LDL target than participants from Europe. The numbers of antihypertensive medications per patient were broadly similar, with slightly more US participants receiving none or one and slightly fewer receiving two, three, or four of these medications, compared with European participants. More US than European participants with prior CVD had decreased eGFR. The percentages of participants with a prior myocardial infarction or angina pectoris also differed significantly between regions (Table 1).

### Participants with HbA1c at target

Among participants without prior CVD, after multivariable adjustment, the odds ratio (OR) for US participants to have HbA1c ≤7.5 % (≤58 mmol/mol) at baseline was 0.475 (95 % confidence interval [CI] 0.316; 0.704) compared to European participants (*P* < 0.001). In the group of participants with prior CVD, the corresponding OR for US participants was 0.762 (95 % CI 0.647; 0.896) (Table 2). With a target ≤7 % (≤53 mmol/mol), the OR for US participants to be at target at baseline was 0.532 (95 % CI 0.260; 1.020) (not significant [NS]) in the no prior CVD group, and 0.839 (95 % CI 0.646; 1.084) (NS) in the prior CVD group.

**Table 2:** Determinants of being at HbA1c target at baseline (HbA1c ≤ 7.5 %), stratified by prior CVD status

	No prior CVD group (*n* = 1013)			Prior CVD group (*n* = 4996)		
	Odds ratio	95 % CI	*P* value	Odds ratio	95 % CI	*P* value
Region (Europe as reference group)	0.475	0.316; 0.704	<*0.001*	0.762	0.647; 0.896	*0.001*
Age (per year)	1.015	0.981; 1.050	0.393	1.032	1.020; 1.043	<*0.001*
Sex (male as reference group)	0.485	0.169; 1.271	0.148	0.834	0.685; 1.011	0.065
Diabetes duration (per year)	0.976	0.951; 1.000	0.055	0.976	0.967; 0.986	<*0.001*
Race (white as reference group)						
Asian	0.970	0.139; 4.258		0.598	0.257; 1.223	
Black	1.691	0.672; 4.244	0.718	1.069	0.780; 1.452	0.266
Other	1.358	0.195; 6.005		0.604	0.258; 1.248	
Pre-trial diabetes treatment (none, diet as reference group)						
Insulin + OADs	1.080	0.475; 2.710		0.800	0.586; 1.102	
Insulin only	0.956	0.272; 3.215	0.543	0.532	0.349; 0.805	<0.001
OADs only	1.365	0.629; 3.312		1.248	0.930; 1.693	
BMI (18–25 as reference group) (kg/m^2^)						
25–30	0.628	0.286; 1.446		1.220	0.871; 1.730	
30–35	0.788	0.369; 1.780	0.235	1.010	0.723; 1.427	0.049
35–40	0.619	0.273; 1.464		1.036	0.729; 1.488	
>40	1.082	0.476; 2.575		0.824	0.561; 1.219	
Creatinine, per μmol/L	0.966	0.915; 1.015	0.179	0.998	0.993; 1.002	0.288
ACR, per mg/mmol	1.002	0.997; 1.005	0.421	1.000	0.999; 1.002	0.471
eGFR, per mL/min/1.73 m^2^	0.967	0.931; 1.000	0.048	0.992	0.986; 0.998	*0.007*

Logistic multivariable regression modelling the probability of being at HbA1c target, adjusted for: age, sex, diabetes duration, race, pre-trial diabetes treatment, BMI category and renal function (creatinine, ACR, eGFR)

*ACR* albumin:creatinine ratio, *BMI* body mass index, *CI* confidence interval, *CVD* cardiovascular disease, *eGFR* estimated glomerular filtration rate, *OAD* oral antidiabetic drug

Overall *P* values relating to the significance of that factor

### Participants with BP at target

After adjustment for age, sex, race, diabetes duration, BMI class, number of hypertension medications, renal function, and well as micro- and macro-albuminuria, US participants were more often at target BP than participants from Europe, irrespective of their CVD history (no prior CVD OR 1.662; 95 % CI 1.150;2.402; *p* = 0.007; prior CVD 1.314; 95 % CI 1.136; 1.520; *P* < 0.001) (Table 3).

**Table 3:** Determinants of being at BP target (Europe <140/80 mmHg, USA <130/80 mmHg) at baseline stratified by prior CVD status

	No prior CVD group (*n* = 1013)			Prior CVD group (*n* = 4996)		
	Odds ratio	95 % CI	*P* value	Odds ratio	95 % CI	*P* value
Region (Europe as reference group)	1.662	1.150; 2.402	*0.007*	1.314	1.136; 1.520	<*0.001*
Age (per year)	0.959	0.928; 0.990	*0.009*	0.992	0.982; 1.002	0.115
Sex (male as reference group)	0.823	0.341; 1.707	0.641	1.070	0.901; 1.270	0.438
Diabetes duration (per year)	1.020	0.998; 1.042	0.082	1.003	0.995; 1.012	0.472
Race (white as reference group)						
Asian	1.758	0.457; 6.795		0.880	0.483; 1.560	0.064
Black	1.070	0.517; 2.318	0.604	0.724	0.551; 0.946	
Other	0.442	0.063; 1.987		0.621	0.304; 1.189	
BMI (18–25, reference group) ( kg/m^2^)						
25–30	1.217	0.577;2.677		0.889	0.655; 1.212	
30–35	0.913	0.436;1.998	*0.009*	0.793	0.587; 1.077	*0.008*
35–40	0.511	0.229;1.177		0.693	0.503; 0.957	
>40	0.650	0.283;1.533		0.623	0.442; 0.879	
Number of antihypertensive medications						
0 (reference group)						
1	1.142	0.698; 1.897		0.918	0.693; 1.221	
2	0.716	0.415; 1.244	0.096	1.010	0.772; 1.326	<*0.001*
3	0.650	0.333; 1.256		0.648	0.477; 0.881	
4	1.024	0.360; 2.670		0.646	0.424;0.975	
Creatinine, per umol/L	0.986	0.942; 1.021	0.488	1.004	1.000; 1.008	0.058
ACR, per mg/mmol	0.992	0.983; 0.999	0.013	0.994	0.992; 0.996	<*0.001*
eGFR,per mL/min/1.73 m^2^	0.985	0.956; 1.007	0.239	0.998	0.992; 1.003	0.334
Micro-albuminuria	0.895	0.628; 1.275	0.538	0.947	0.774; 1.155	0.592
Macro-albuminuria	0.918	0.507; 1.635	0.775	1.562	1.060; 2.287	0.024

Logistic multivariable regression modelling the probability of being at BP target, adjusted for: age, sex, diabetes duration, race, BMI category, number of antihypertensive medications, renal function (creatinine, ACR, eGFR), and micro- and macro-albuminuria

*ACR* albumin:creatinine ratio; *BMI* body mass index; *BP* blood pressure; *CI* confidence interval; *CVD* cardiovascular disease; *eGFR* estimated glomerular filtration rate

Overall *P* values relating to the significance of that factor

### Participants with LDL at target

US participants with no prior CVD were more often at LDL target at baseline, with a multivariable adjusted OR of 1.616 (95 % CI 1.116; 2.355; *P* = 0.011) compared with European participants. In contrast, in participants with prior CVD, US participants were less likely to be at LDL target than European participants (OR 0.248; 95 % CI 0.212; 0.290; *P* < 0.001). Not surprisingly, statin use was an independent determinant for achieving LDL target, irrespective of whether it was prescribed as primary or secondary CVD prevention (Table 4).

**Table 4:** Determinants for being at LDL target at baseline (Europe <100 mg/dL, USA <100 mg/dL for no prior CVD and <70 mg/dL for prior CVD group)

	No prior CVD group (*n* = 1013)			Prior CVD group (*n* = 4996)		
	Odds ratio	95 % CI	*P* value	Odds ratio	95 % CI	*P* value
Region (Europe as reference group)	1.616	1.116; 2.355	0.011	0.248	0.212; 0.290	<*0.001*
Age (per year)	1.038	1.006; 1.072	0.020	1.012	1.001; 1.023	0.032
Sex (male as reference group)	0.542	0.311; 1.009	0.053	0.535	0.444; 0.644	<*0.001*
Diabetes duration (per year)	1.011	0.989; 1.035	0.330	1.004	0.995; 1.013	0.358
Race (white as reference group)						
Asian	0.632	0.136; 3.479		1.226	0.664; 2.275	
Black	0.710	0.371; 1.339	0.633	0.773	0.581; 1.023	0.226
Other	1.53	0.313; 8.783		0.759	0.389; 1.451	
BMI (18–25, reference group) (kg/m^2^)						
25–30	0.571	0.239; 1.298		1.029	0.735; 1.438	
30–35	0.836	0.353; 1.884	0.224	1.083	0.778; 1.503	0.472
35–40	0.879	0.361;2.049		1.105	0.782; 1.560	
>40	0.935	0.375; 2.246		1.287	0.891; 1.856	
Statin use (no as reference)	4.669	3.352; 6.550	<*0.001*	4.276	3.526; 5.202	<*0.001*
Aspirin use (no as reference)	1.055	0.750; 1.482	0.759	1.369	1.174; 1.597	<*0.001*
Creatinine, per umol/L	1.007	0.982; 1.038	0.607	0.995	0.991; 1.000	0.034
ACR, per mg/mmol	0.999	0.995; 1.003	0.485	0.999	0.997; 1.000	0.017
eGFR per mL/min/1.73 m^2^	1.006	0.993; 1.025	0.360	0.994	0.988; 1.000	0.036

Logistic multivariable regression modelling the probability of being at LDL target, adjusted for: age, sex, diabetes duration, race, BMI category, statin use, aspirin use, and renal function (creatinine, ACR, eGFR)

*ACR* albumin:creatinine ratio, *BMI* body mass index, *CI* confidence interval, *CVD* cardiovascular disease, *eGFR* estimated glomerular filtration rate, *LDL* low-density lipoprotein

Overall *P* values relating to the significance of that factor. Stratified by prior CVD status

## Discussion

This study identified relevant differences in baseline cardiovascular risk profiles between European and US participants in the LEADER trial. These differences should be considered in the analysis and reporting of the trial results.

LEADER aims to formally assess the cardiovascular safety of liraglutide, as reflected in the number of cardiovascular events. The trial population consists of people with/without history of CVD. The large number of subjects, as well as the number of study sites, will ensure the results reflect wide clinical practice, and thus the robustness of the primary results. However, whether liraglutide is noninferior to placebo [1] in subgroups will at least partially depend on the baseline risk of the participants. The European and US populations in LEADER differed in many factors related to cardiovascular risk, including race, BMI, HbA1c, SBP and DBP, ventricular dysfunction, and, in the group without prior CVD, albuminuria. In some aspects, US participants may have a lower cardiovascular risk because of their substantially lower BP level and lower prevalence of left ventricular dysfunction, but this could be counteracted by higher cardiovascular risk because of higher BMI and HbA1c. The baseline differences identified should be explored in relation to the overall trial results, once reported. Specifically, testing for heterogeneity of the treatment effect and interactions between subgroups would be needed to evaluate whether these differences impact upon the generalizability of the overall trial findings. Thus, these baseline findings should be interpreted with caution, but may inform additional analyses. Calculating an individual’s 10-year CVD risk in people without prior CVD may also help identify which patients benefit most from the treatments under study [15, 16].

Regional differences are not unique to LEADER: they probably exist in all global trials, and in cardiovascular outcome trials in diabetes in particular. The analysis in this study provides more detail on potential relevant differences, and hence will support interpretation of the final results. The implications of these data, which show differences related to baseline cardiovascular risk management between US and European participants, may also be relevant in shaping future trial designs with similar populations. Such trial designs will need to account for factors pertaining to cardiovascular risk.

Strengths of this study include the large patient groups involved and investigation of the role of the ‘region’, independently from patient-related anthropometric and disease-related factors. Region, i.e. Europe or US (a cultural-, socio-economic-, and healthcare-related characteristic), was the most important factor associated with participants achieving HbA1c, BP, and LDL targets. Many of the baseline differences between the US and Europe appear driven by variation in regional targets for cardiovascular risk management. However, the role of ‘region’ may not only be determined by guidelines and physician prescription patterns, but also the sites that enrolled patients, the way they did so, and patient‒physician relationships. A recent systematic review and meta-analysis of randomized controlled trials demonstrated that intensive glycemic control in patients with T2D was associated with increases in all-cause mortality, cardiovascular mortality, and severe hypoglycemia in study populations from North America compared with the rest of the world [17]. Its authors emphasized the need for further investigation into these findings [17]. The choice of participating centers can also influence the generalizability of trial results. For example, among >55,000 high-risk patients presenting with non-ST-segment elevation acute coronary syndrome at 443 US hospitals, 1397 (2.5 %) patients were enrolled in a clinical trial. The latter were more often cared for by cardiologists at hospitals capable of catheter-based and surgical revascularization—which might explain the high rates of revascularization among clinical trial patients [6]. Such discrepancies in care and patient characteristics would suggest that the data from this and similar cardiovascular studies cannot be extrapolated to the broad type 2 diabetes population. In the global Trial to Evaluate Cardiovascular Outcomes after Treatment with Sitagliptin (TECOS), regional differences were noted, with lower BP and cholesterol levels in US participants, compared to participants both from western and eastern Europe. Treatment target achievement was also associated with the type of prior vascular disease [18]. Therefore, region-specific data about the centers that enrolled patients (for example, academic sites, rural sites etc.), and region-specific loss-to-follow-up data would enable readers to judge the applicability of the trial results for their practice population.

LEADER was undertaken to assess the cardiovascular safety of liraglutide. A large set of exploratory subgroup analyses have been pre-specified to assess the effect of (among others) sex, BMI, region, cardiovascular risk, chronic heart failure, and severe chronic renal failure on the primary composite endpoint [1]. The present study demonstrates the relevance of these planned subgroup analyses and of evaluating any heterogeneity in outcomes. In this respect, it could also be relevant to present detailed data about sites of enrolment and loss to follow-up. Finally, patient-level meta-analysis of the CVD trials underway would allow for deeper exploration of these issues, as sufficient sample size of many subgroups would be available [19].

## Conclusions

This study demonstrated important baseline differences between US and European participants in the LEADER trial. These differences may be the result of regional variations in targets for cardiovascular risk factor management. These factors should be considered in the analysis and reporting of the trial results and may also be relevant for potential post hoc investigations.

## Abbreviations

ACR: albumin:creatinine ratio
ADA: American Diabetes Association
BMI: body mass index
BP: blood pressure
CI: confidence interval
CVD: cardiovascular disease
DBP: diastolic blood pressure
ECG: electrocardiogram
eGFR: estimated glomerular filtration rate
HbA1c: glycated hemoglobin
LDL: low-density lipoprotein
LEADER: Liraglutide Effect and Action in Diabetes: Evaluation of Cardiovascular Outcome Results
NS: not significant
NYHA: New York Heart Association
OR: odds ratio
SBP: systolic blood pressure
T2D: type 2 diabetes
TECOS: Trial to Evaluate Cardiovascular Outcomes after Treatment with Sitagliptin
